# Exploring the psychological experience of novice nurses in stomatological hospitals in China: a phenomenological study

**DOI:** 10.1186/s12912-024-01879-z

**Published:** 2024-04-01

**Authors:** Jian Liu, Tiantian Lu, Yan Li, Hongyuan Dai, Li Li

**Affiliations:** Nanjing Stomatological Hospital, Affiliated Hospital of Medical School, Institute of Stomatology, Nanjing University, Nanjing, China

**Keywords:** Novice nurse, Stomatological hospital, Psychology, Qualitative study, Phenomenological research

## Abstract

**Background:**

At the onset of their professional journey, novice nurses often undergo a multifaceted psychological experience as they transition from theoretical knowledge to clinical practice, potentially impacting their development of professional identity. However, limited research has been conducted on the psychological aspects pertaining to newly graduated nurses in stomatological hospitals in our country.

**Methods:**

The phenomenological method and semi-structured interviews were used in this study, and the sample size of the interview was purposive sampling method. A semi-structured virtual interview was conducted with 21 new nurses in the department of stomatology. Colaizzi’s analysis method was used to analyse the interview data.

**Results:**

Based on Kramer’s reality shock theoretical framework and analyzing interview data, this study extracted the psychological experiences of novice nurses during their first year of employment across four distinct stages. The four stages include: cheerful period, frustration period, adjustment period and competency period. Six themes and nine sub-themes were derived from the four period.

**Conclusion:**

Due to the lack of professional knowledge, novice oral nurses will experience a series of complex positive and negative emotions at the beginning of their career. Through the research, the training of oral specialty theory, good psychological counseling and peer support can improve their participation in oral outpatient nursing. At the same time, the establishment of oral care quality assurance system and the improvement of oral care higher education in our country will become the focus of future research.

## Background

According to statistics, there has been a recent increase in the number of patients with oral diseases in China, resulting in a total of 703 million cases of dental caries [1], malocclusion, pulpitis, and other oral conditions [2]. The work of oral nurses is not limited to daily oral treatment assistance, but also involves oral health education, preventive health care, oral imaging technology, instrument disinfection and sterilization, four-hand operation cooperation, and oral disease management and research. With the increasing public awareness of oral health and the growing demand for oral medical services, the demand for oral nurses continues to increase. Dental nurses play a crucial role as integral members of the medical service team in the field of oral health care. However, studies [3] have indicated that most dental clinic nurses receive training primarily focused on general nursing rather than specialized oral care. In clinical practice, these student nurses typically undergo rotations lasting over eight months at grade-A tertiary general hospitals [4]. Novice oral nurses lack systematic basic knowledge of oral nursing specialty and related practical courses before entering the oral outpatient nursing work. At the beginning of the work, the new nurse is enthusiastic about the work. After entering the workplace for some time, they feel anxious and helpless, and crave help from colleagues and care managers. At the same time, due to the increase in work intensity and responsibility, the novice nurses will become less confident and even increase the turnover rate [5, 6]. Therefore, adopting a phenomenological perspective for this study involved conducting in-depth interviews with newly employed nurses who did not specialize in oral nursing but had recently joined stomatological hospitals. The aim was to extract their psychological experiences during the transitional stage [7], identify strategies for stress relief and coping mechanisms while providing valuable insights for enhancing professional development and management practices among nurses within stomatological hospitals.

## Methods

### Objective of the study

The purpose of this study was to explore the psychological experience of novice nurses in different stages of early work in stomatological hospitals, and to provide evidence for future training of stomatological nurses.

### Participants

Purposive sampling method was used to select 21 newly recruited nurses from different regions and different grades of stomatology hospitals for interview. They are going through the early stages of their career development,with less than one year of work experience. The inclusion criteria were as follows: (I) Graduation with a nursing major and possession of a valid nurse practicing certificate; (II) Engagement in dental specialist outpatient nursing duties; (III) Less than one year of working experience; (IV) Provision of informed consent and voluntary participation in this study. Exclusion criteria included: (I) Taking sick leave or being on sick leave for more than three months; (II) Transferring to non-oral outpatient nursing positions. The sample size was determined based on information saturation, ensuring no new topics emerged.

## Data collection

A semi-structured interview guide (Table 1) was used to collect information through face-to-face interviews. This interview guide was based on a thorough literature review [8] and extensive discussions within the research group. The interview guide was then refined through consultations with two dental specialists, two dental outpatient nursing managers, one dental nurse, and pre-interviews with five novice nurses. All the interviews were conducted by a postgraduate nursing student who was trained in qualitative research. A research assistant played an auxiliary role which included recording the interviews. The interview took place in the nurses’ lounge and lasted 30 to 40 min.The interviews were recorded by a whole process of synchronous recording, notes, and timely reminiscence during the interview, and participants’ views were clarified and confirmed to ensure accuracy. The interview times ranged from 30 to 40 min. The interviews were conducted in the participant’s language (Chinese). At the end of each interview, two Doctor of Nursing who are familiar with the disciplinary background and professional terms were invited to translate the interview content into English to ensure the accuracy of the translation.We stopped when the analysis reached data saturation: the interview has not added any new information to the data collection process. The sample size was saturated when the information appeared repeatedly and there was no new content or topic. The study did not repeat the interviews.

**Table 1:** Semi-structured interview guide

Semi-structured interview guide
1. Why did you choose to work in dental outpatient care?
2. According to your current circumstances, what are the challenges encountered in the professional domain?
3. What are the distinguishing characteristics between clinical nursing and oral outpatient nursing in terms of their scope and practice?”
4. In what ways do you anticipate nursing managers providing assistance in your future professional endeavors?

## Data analysis

Throughout the analysis, two researchers independently reviewed and coded the interview transcripts, identifying initial themes and subthemes. This study adopts the Colaizzi descriptive analysis framework [9] divided into seven analytical steps. (1) Reading of interviews by two researchers who reread transcripts several times to become immersed in the data. (2) Identification and coding of significant statements related to psychological concerns in novice nurses.(3) Extracting meaningful fragments through team discussions. (4) Organizing each significant statement into meaningful units and subthemes into major themes. (5) Linking themes closely to research phenomena and detailing them. (6) Identifying similar ideas and deleting them. (7) Providing feedback on the results to participants to ensure the authenticity of the content. To enhance reliability and agreement, a third researcher was involved in developing credibility around the themes and addressing any discrepancies between the initial coders.

## Ethical considerations

The Ethics Committee of the Stomatology Hospital Affiliated to the School of Medicine of Nanjing University provided an ethical review. All participants signed an informed consent document before the interview. Additionally, participants were informed that their participation in the study was voluntary and the information gathered from the interviews would be recorded and used only for scientific purposes. To protect the privacy of interviewees, the interviews were presented anonymously, and names were replaced with numerical identifiers.

## Rigour

Rigour was ensured by following the criteria for trustworthiness: credibility, confirmability, dependability and transferability. During the interview, the researcher asked questions according to the interview outline, listened carefully, asked questions, retold and summarized in a timely manner, and kept an objective attitude without inducing, evaluating or easily interrupting the interviewees’ statements,and and this methodology strengthened the overall credibility of the study. All of the participants confirmed that the results resonated with their experiences, and no new feedback had developed in their practice since the initial interviews. All authors took part in all analytical stages. The condensed text was discussed until consensus was reached on the analysis and the formulated themes. This strengthens the dependability of the study, together with a clear description of the research process. The results are presented with participants’ quotes to facilitate external judgement and to consider transferability of results to other nursing contexts.

## Findings

A total of 21 novice nurses participated in the interview and their demographic information is shown in Table 2. There were 18 female nurses and 3 male nurses, ranging in age from 20 to 23 years. The themes in the interviews are presented in Table 3.

**Table 2:** Socio-demographic characteristics of participants (*N* = 21)

Code	Age	Gender	Reasons for working in the stomatological hospitals
N1	23	Female	2
N2	21	Female	1, 2, 5
N3	22	Female	2, 3, 5
N4	20	Female	1, 3, 4
N5	22	Female	2, 5, 6
N6	23	Female	1, 2
N7	21	Female	1, 2, 5, 6
N8	21	Female	1, 2, 3, 6
N9	22	Female	3
N10	22	Male	2, 6
N11	23	Female	2
N12	23	Female	2, 3, 5, 6
N13	20	Female	1, 4
N14	23	Male	3, 4
N15	22	Female	4
N16	22	Female	3
N17	23	Female	3, 4, 5
N18	22	Female	1, 4
N19	22	Female	1, 2, 5, 6
N20	21	Male	3, 4
N21	22	Female	1, 2

**Reasons for working in the stomatological hospitals**: 1 = Clean working environment; 2 = Simple interpersonal relationship; 3 = Personal interest; 4 = Good prospects for development; 5 = High income; 6 = Low working pressure

**Table 3:** Summary of themes and sub-themes

Period	Themes	Sub-themes
The cheerful period	Pleasant and relaxation	Keeping a positive attitude towards learning Avoiding nurse-patient conflict
The frustration period	Self-doubt Helpless Frustration	Peer support Professional support Need to maintain self-esteem
The adjustment period	Relieved	Managing negative emotions Making up for the shortcomings
The competency period	Self-affirmation	Cognitive reappraisal Transform knowledge

## The cheerful period: enter the new work environment feel temporary pleasant and relaxation

### Theme 1: pleasant and relaxation

#### Keeping a positive attitude towards learning

All interviewees reported that oral outpatient nursing was less physically demanding than clinical nursing for the same amount of time. At the same time, because the night shift in the oral clinic decreased or even some of them did not have night shift, they all indicated that the daily self-management time increased, and they were more willing to accept nursing work without night shift. They are eager to learn new things.The participants provided the following accounts of their experiences:

“Before that, I worked as an intern in the intensive care unit of a Grade III general hospital, where I had to turn over for patients every two hours. I was very nervous and tired every day, because I did not want to continue working night shifts, so I want to learn about oral care”. (N5)

“I am very interested in every nursing operation, hope to master as soon as possible, and want to learn new knowledge every day”.(N6).

#### Avoiding nurse-patient conflict

The working environment of stomatology hospital is different from that of general hospital, the oral clinic is relatively clean and quiet, and doctors are responsible for the main treatment and communication work. Nurses need to cooperate with doctors in the process of diagnosis and treatment to prepare materials before diagnosis and treatment, four-hand operation, delivery of goods, mixing materials, disposal of materials, and health education for patients, etc., which avoids long-term direct contact between nurses and patients and reduces nurse-patient conflicts. The working environment of general hospital is noisy and busy, which is easy to cause the contradiction between nurse and patient. Participants shared their experiences in this regard:

“There is no need to directly face the patient, there is no need to explain to the patient before doing the operation, only cooperate with the doctor to pass the instrument, do their own work, and never conflict with the patient”.(N18).

“I am very friendly to every patient, working in the dental clinic smile is essential”.(N4).

## The frustration period: the expectation of the new role is very different from the daily work and lack of security

### Theme 2: self-doubt

#### Peer support

With the passage of time, their own high demands on the work and often frustrated in the outpatient nursing work, the new nurses have become less and less confident. They crave understanding and support. Participants shared their experiences as follows:

“When mixing materials, I did not have a good grasp of the nature of the materials, and each doctor had different preferences. Sometimes I would confuse their preferences, which made them dissatisfied, and I became less and less confident. After the work, I will talk to my good friends, they show understanding and will give me support”.(N7).

“My friends tell me you’ll do a lot of operations. For example, intravenous infusion, enema, sputum aspiration and other operations. It’s just not useful in your current field of work”.(N8).

### Theme 3: helpless

#### Professional support

Novice nurses encounter setbacks in the workplace, feeling helpless and eager to seek professional help. At the same time, they tend to have low career achievements due to lack of professional guidance and work mistakes. However, compared with the new nurses who entered the general hospital, they have been more competent because of the systematic training of clinical nursing professional knowledge.

“I want to learn some professional knowledge about oral care, which will improve my confidence. So I hope that nursing managers can increase their training in oral knowledge and oral nursing practice skills”.(N18).

“I was nervous at work, afraid of doing it wrong, and I wanted someone (nursing managers) to guide me in oral care. If I master the professional knowledge, I may not be nervous in the future work”.(N7).

### Theme 4: frustration

#### Need to maintain self-esteem

After the short enthusiasm for the new environment, with the increase of work intensity and the requirements of job duties, novice nurses who have just entered the hospitals will often feel uneasy and even fall into frustration due to their lack of oral care expertise and weak practical operation. Participants shared their experiences as follows:

“Sometimes I am nervous and afraid of making mistakes in my work, A few times I’ve seen doctors shake their heads disdainfully at me because I’ve made a mistake in my work”.(N14).

“I felt that it was too different from the clinical, I felt that I was out of place, and the operational skills I knew before were less useful here”.(N15).

## The adjustment period: the career development transitioned from a state of confusion to gradually adapting

### **Theme 5: relieved**

#### Managing negative emotions

At this stage, nursing students realize their negative emotions in the work, begin to adjust their mentality.

“When I’m feeling down, I take a deep breath and whisper to myself to be confident”.(N1).

“I will think of something pleasant and try not to bring negativity into my work”.(N6).

#### Making up for the shortcomings

Novice nurses begin to accept their shortcomings in ability and strive to enrich themselves, and gradually adapt to the nursing work in the oral clinic. Participants shared their experiences as follows:

“Like the previous internship, I will prepare a small book, and record in time what I don’t understand in my usual work. After work, I will consult the information or consult my colleagues and head nurses”.(N19).

“When I get home from work, I start reading oral care books, sometimes studying until the wee hours of the morning. I don’t want to fall behin’’.(N3).

## The competency period: be able to put your role in perspective and be competent in clinical work

### Theme 6: self-affirmation

#### Cognitive reappraisal

Novice nurses with improved abilities in all aspects can independently carry out nursing operations, and when they encounter problems, they are no longer afraid and uneasy, and begin to think about how to use existing resources to change the status quo. They are eager for long-term development, and strive to become a dental specialist nurse in order to better show their value in work. Participants shared their experiences as follows:

“At work, I have a cooperative relationship with doctors, but we have different division of labor. Whether it is clinical work or oral clinic work, it is a process of continuous learning and accumulation of knowledge. I believe that I can become a qualified and competent nurse in any field”. (N21)

#### Transform knowledge

By strengthening the learning of oral nursing professional knowledge and professional skills, new nurses will combine the previous knowledge with the new knowledge and apply it to the field of work.

“After all, I was in clinical practicum, and the doctor praised me for my strong aseptic concept when I assisted the doctor with the surgery. I found that clinical and oral outpatient work have many similarities, but we need to transform and accurately apply it to the usual outpatient work”.(N11).

## Discussion

This study found that after the newly graduated nurses worked in the stomatological hospitals, the outpatient work mode reduced the damage to their body caused by high-intensity night shift, and the change of working environment reduced the nurse-patient conflict, both of which alleviated the physical and mental pressure of such nurses. It shows that improving the working environment can relieve the working pressure of nurses. However, in terms of work content, they are prone to frustration due to their proficiency in oral nursing when they first enter the job due to their weak knowledge base in oral nursing. At this time, nurses will have negative emotions, which requires them to be good at regulating their emotions.Secondly, the nurses in the dental clinic are mainly engaged in nursing cooperation beside the chair, and the relationship between nurses and doctors has changed from cooperation to subordination, and the sense of professional achievement has decreased. Therefore, it is suggested that nursing managers should strengthen the training of the basic knowledge and practical ability of the new oral nurses after they enter the clinical work. Make practical learning plans and training plans to guide the career planning of nurses. At the same time, in terms of psychology, psychological experts should be used to guide the psychology of novice nurses, which should be different from person to person. To help and support novice nurses in work and life, improve the status and importance of nurses in oral diagnosis and treatment, and finally realize the high-quality development of oral care.

### Improving the working environment

Studies have shown that [10] poor working environment, uncooperative service objects and overloaded workload tend to cause nurses to be in a state of long-term high pressure. Long-term high pressure can lead to anxiety, empathy fatigue, headache and other sub-health [11], which also enhances nurses’ turnover intention. In addition, greater work pressure will not only affect the physical and mental health of nurses [12], but also pose a threat to the life safety of patients. This study shows that the working environment in the dental clinic is more conducive to nurses’ physical and mental relaxation than the clinical working environment. The most direct and obvious impact is to protect nurses’ sleep and reduce nurse-patient conflicts. Among them, all interviewees mentioned that within the same working hours, oral outpatient nursing work consumes less physical energy, and self-perception of oral outpatient working environment is better. This is consistent with the survey results of Wang Qian [5] et al. Therefore, working environment, work load and good nurse-patient relationship are more conducive to nurses’ involvement in work, and are also one of the factors for nurses to choose jobs. The stomatological hospitals should pay attention to the working environment and workload of nurses, conduct on-the-job skills and theoretical knowledge training for nurses, and take corresponding measures to improve the working environment and timely adjust the allocation of human resources, so as to alleviate the physical and mental fatigue of nurses and reduce the turnover rate of nurses.

### Regulating your emotions

According to the emotional regulation process model of Campos [13], the purpose of individual emotional regulation is to choose socially acceptable responses to coordinate inconsistent conflicts in daily interactions. If not solved in time, the negative psychology in interpersonal communication is likely to cause individuals to have distress and avoidance behaviors in social activities [14, 15]. In this study, with the increase of work intensity over time, nurses in the transition period felt more conflict between ideal and reality. On the one hand, due to the lack of theoretical knowledge and practical skills in stomatology, novice nurses often frustrated in nursing work, which leads to the reduction of professional achievement; On the other hand, there are studies [16] showing that nurses will take doctors as the reference objects for social comparison in terms of career promotion, social status and treatment. The more unfairness they feel, the more negative emotions they will have. Therefore, for the psychological conditions faced by novice nurses, medical institutions should invite psychological experts to psychological counseling them to help them get out of negative emotions as soon as possible. Help them re-examine their role and correctly understand their responsibilities and importance in the work [17]. It is understood that although the working environment and nursing operation of the dental clinic are different from the clinical clinic, the essence of nursing work is patient-centered, rather than focusing on a certain specialty. Different professional and technical personnel need to collaborate with each other to provide better service to patients.

### Positive learning attitude

In traditional Chinese ethics, people attach great importance to the cultivation of learning attitude. Some studies [18, 19] show that positive learning attitude helps cross-professional nursing students master professional knowledge, integrate into team work more quickly and improve critical thinking of nursing students. In this study, some of the nurses in the dental clinic during the transition period had clinical work experience, or even had rich work experience and status. However, due to the change of the working environment, the weak theoretical knowledge and practical operation ability of stomatology will make them feel uncomfortable. However, after self-emotional management and psychological construction, they have given up the praise of the past honor, maintain a positive learning attitude in daily work, and strive to enrich themselves to fill the vacancy in oral oral care specialist theoretical knowledge. Therefore, it is suggested that nurses should maintain a positive learning attitude at any stage of their work to enrich their professional knowledge. At the same time, nursing managers should also pay attention to the cultivation of nurses’ learning attitude.

### Improving the ability of knowledge transfer

The ability to acquire and transform knowledge [20] is more important than knowledge itself, and to achieve the integration of knowledge and action requires improving one’s own knowledge transformation ability. SECI [21], a knowledge transformation model proposed by Ikujiro Nonaka, holds that tacit knowledge and explicit knowledge can transform each other. In this study, clinical nurses based on clinical nursing knowledge applied strong aseptic concept to oral outpatient nursing work, and also learned oral medicine knowledge in the work. As a branch of nursing, oral nursing adds the basic course of oral medicine on the basis of nursing, which is the cross specialty of nursing and oral medicine. Therefore, it is suggested that nurses should quickly transform the relevant theoretical knowledge and practical experience in nursing, oral care and oral medicine into their work field, and treat their work and patients with a proactive attitude [22]. Learn to use their own knowledge, experience, communication and other advantages combined with the existing environment, strengthen the ability of knowledge transformation, so that no matter how the working environment changes, as a nurse can quickly qualified for new posts. Just as scholar Han Bin [23] said, “Only by targeting the needs of patients and providing professional guidance can the nursing specialty outpatient clinics have vitality”.

### Study limitations

The study was limited by its use of a single data source, as participants were recruited from only two dental specialty hospitals and were not generalized nationwide. So it may not be generalizable to other nurses.

### Implications for practice

The study provides evidence for improving the adaptability of new graduates to work more quickly.Nursing managers should strengthen the training of the newly graduated nurses on the theoretical knowledge of the specialty, and at the same time, they should also provide psychological counseling to such nurses to help them face the work more actively and grow into an excellent nurse quickly.

## Conclusion

Through in-depth interviews with 21 novice dental nurses, the objective of this qualitative study was to gain insight into the psychological experience of novice nurses in the stomatological hospitals and to extract their psychological experiences during the transitional stage, identify strategies for stress relief and coping mechanisms while providing valuable insights for enhancing professional development and management practices among nurses within stomatological hospitals. Through the research, it is found that the dental hospital has some deficiencies in the training of the theoretical knowledge and practical operation ability of the dental novice nurses. In the future, it is suggested that scholars and dental hospitals develop feasible training programs for new dental nurses. In the aspect of psychological counseling, establish a professional organization to carry out psychological counseling for novice nurses. It is suggested that China should improve the specialized education of oral nursing, and form a specialized nursing education system with higher nursing education as the mainstream.

## Data Availability

The datasets generated and/or analysed during the current study are not publicly available, but are available from the corresponding author on reasonable request.

## References

[1] Consumer Healthcare Industry Depth. Industry Atlas– 220901 (58 pages).

[2] Xia YA, Cao YX, Yu XY. A case report and literature review of implant repair of severe class III alveolar bone [J/OL]. Journal of Ji Nan university (natural science and medicine edition),2023,09(02):1–6.

[3] Yin YL, Han JN, Fu L (2022). Current situation and enlightenment of training oral nursing professionals in China [J]. Chin J Nurs Educ.

[4] Wang Q, Mandew, Shi Yue Xian (2022). Investigation on job involvement and influencing factors of outpatient nurses in Stomatology hospital [J]. J Nurs.

[5] Zhu YQ, Zhang YY, Zhang YQ (2023). Research progress of transition impact of newly graduated nurses [J]. China Nurs Adm.

[6] Huang YX. Study on the impact Degree of nursing undergraduates’ transformation and its influencing factors [D]. Chongqing Medical University; 2022.

[7] Mo YY, Su ZQ, Li YF (2022). Factors influencing the working mental state of dental nurses [J]. Nurs Pract Res.

[8] Han YT, Wu YF, Hou LL (2023). Application of psychological course based on reality impact theory in the training of new nurses [J]. Shanghai Nurs.

[9] Webb C. Information point: Colaizzi’s framework for analysing qualitative data[J].Clin Nurs,1999, 8(5):576. 10786530

[10] Maher L, Dertadian G (2018). Qualitative research. Addiction.

[11] Peng J, Li D, Zhang Z (2016). How can core self-evaluations influence job burnout? The key roles of organizational commitment and job satisfaction [J]. J Health Psychol.

[12] Wei LW, He QH, Wang MM (2022). The relationship between work time stress and mental health of graduate students: the mediating effect of sleep quality and the moderating effect of mental flexibility [J]. Chin J Health Psychol.

[13] Zhang HL, Huo XN, Zhao ZY (2022). The mediating effect of health promoting lifestyle on job stress and sleep quality in nurses [J]. Chin J Health Psychol.

[14] Campos JJ, Mumme DL, Kermoian R (1994). A functionalist perspective on the nature of emotion [J]. Monogr Soc Res Child Dev.

[15] Xue J, Hong JW. Withdrawal self-rescue under burden: An Analysis of Young users’ social media burnout Behavior based on Rooted theory [J]. News Writ, 2022, (08): 70–83.

[16] Zhang GJ, Liu HL. Expression of loneliness in social media: an analysis of the advantages and disadvantages of revealing negative emotions and seeking support [J]. Friends Editor, 2022, (09): 77–81.

[17] Dai JH (2014). Investigation and analysis of professional identity of college nursing students [J]. Nurs Pract Res.

[18] Gross JJ (2002). Emotion regulation: affective, cognitive, and social consequences [J]. Psychophysiology.

[19] Lau PS, Wu FK. Emotional competence as a positive youth development construct: a conceptual review [J]. Scientific World Journal, 2012, 2012: 975189. 10.1100/2012/975189 PMC3361327 22666176

[20] Lundell Rudberg S, Lachmann H, Sormunen T (2023). The impact of learning styles on attitudes to interprofessional learning among nursing students: a longitudinal mixed methods study [J]. BMC Nurs.

[21] Jiang YC (2014). Dynamic demand and capability generation of knowledge construction growth [J]. Inform Theory Pract.

[22] Farnese ML, Barbieri B, Chirumbolo A (2019). Managing knowledge in Organizations: a Nonaka’s SECI Model operationalization [J]. Front Psychol.

[23] Dai P, Zhang JM (2021). Factors influencing the quality of humanistic care in dental outpatient nurses [J]. Nurs Pract Res.

